# Patient-self-reported history of restraint among 17-year-olds: a retrospective study of records by non-specialist dentists in the public dental service in Hordaland, Norway

**DOI:** 10.1007/s40368-022-00710-0

**Published:** 2022-05-10

**Authors:** R. S. Aarvik, E. J. Svendsen, M. L. Agdal

**Affiliations:** 1 Institute of Health and Society, Faculty of Medicine, University of Oslo, Oslo, Norway; 2 Oral Health Centre of Expertise in Western Norway, Bergen, Norway; 3 Department of Nursing and Health Promotion, Oslo Metropolitan University, Oslo, Norway; 4 Department of Research, Sunnaas Rehabilitation Hospital, Nesoddtangen, Norway

**Keywords:** Dental records, Behavioural science, Paediatric dentistry, Adolescents, Restraint, Dental fear and anxiety

## Abstract

**Purpose:**

The primary purposes were to examine dental records of Norwegian adolescents’ with and without self-reported history of restraint for information about oral health (DMFT), total scheduled time in the Public Dental Service (PDS) (dental appointments, cancelled and missed appointments), and reluctant behaviour and/or dental fear and anxiety (DFA). Another purpose was to explore their dental records for information recorded by the dentist concerning the use of restraint.

**Methods:**

Data on patient-self-reported history of restraint and DFA were collected in a population-based cross-sectional survey of 17-year-olds in the PDS in Hordaland, Norway, 2019. Patients were divided into two groups: self-reported restraint group (*N*_1_ = 26) and self-reported non-restraint group (*N*_2_ = 200). Data on oral health and dental treatment, total scheduled time of the PDS, reluctant behaviour or DFA, and information on the use of restraint were extracted from the dental records written by non-specialist dentists using a pre-set protocol covering the period from 2002 to 2019.

**Results:**

A total of 206 dental records were analysed. Adolescents with self-reported history of restraint (*n*_1_ = 18) had higher DMFT and greater descriptions of reluctant behaviour and/or DFA, and total scheduled time compared with the self-reported non-restraint group (*n*_2_ = 188). The use of restraint was recorded in the dental records of one patient from the self-reported restraint group and in two patients from the self-reported non-restraint group.

**Conclusions:**

The adolescents with self-reported history of restraint had higher DMFT, higher scheduled time attending the PDS, and had more descriptions of reluctant behaviour and/or signs of DFA compared with the self-reported non-restraint group. The patient records contained limited information concerning restraint, and there were significant discrepancies between patient-self-reported history of restraint and the recording of restraint by the dentist in the patients’ records.

## Introduction

Occasionally children resist dental treatment (Klingberg and Broberg 2007), and their resistance may lead dentists to use restraint during the procedure (Aarvik et al. 2021; Marty et al. 2020). The use of restraint may constitute ethical dilemmas, such as choosing between dental treatments involving the use of restraint or postponing the treatment itself (Aarvik et al. 2021; Marty et al. 2020). Habituating children to dental treatment can be time consuming and patients may experience pain or deterioration of their dental condition if dental procedures are postponed (Aarvik et al. 2021; Romer 2009). The British Society of Paediatric Dentistry provides guidelines on the use of restraint/clinical holding and physical intervention, which likely reflects the current or similar status concerning restraint in relation to children’s dentistry in most European countries (British Society of Paediatric Dentistry 2016). The use of restraint in the dental care of children is restricted to dentists who have undertaken special training concerning the use of restraint in children (British Society of Paediatric Dentistry 2016). In the Norwegian paediatric dental context, physical restraint has been described when a child is held still by a dental health personnel or parents despite the child’s verbal and/or physical resistance, and public non-specialist dentists report that it often occurs in combination with conscious sedation (Aarvik et al. 2021).

The use of restraint during dental treatment may result in fearful behaviour in children (Zhou et al. 2011). In the Public Dental Service (PDS) in Hordaland, Norway, 17- and 9-year-old patients with self-reported history of restraint have significantly higher dental fear and anxiety (DFA) compared with patients without self-reported history of restraint (Aarvik et al. 2022). The strong association between DFA and dental avoidance is well known (Armfield et al. 2007; Fägerstad et al. 2019; Skaret et al. 1999), and the latter has negative consequences for oral health and higher total time use in the PDS (Skaret et al. 1998, 2000; Wang and Aspelund 2009; Åstrøm et al. 2021). In 2009, Wang et al. suggested that children who do not attend their scheduled dental appointments should be considered as risk patients and be offered customised dental care (Wang and Aspelund 2009). Negative dental experiences (range from painful dental treatment to lack of control) in childhood are established as risk factors for developing DFA, and especially painful dental treatment is a frequently mentioned cause of DFA (Klingberg and Broberg 2007; Klingberg et al. 1995; Milsom et al. 2003; Åstrøm et al. 2021). However, the specific experience of restraint and its relation to DFA, dental avoidance, and oral health have received less attention in research.

The Norwegian PDS is required to keep dental records that comprise information that is relevant and necessary to the delivery of healthcare (Health Personnel Act, § 40 1999). Health records are important communication tools for health personnel involved in the patient’s treatment, and are used to promote safety and quality of care, and to reduce the chance of malpractice (Health Personnel Act, § 40 1999). According to Norwegian law, the use of restraint in adults should be documented in health records with its actual and legal reasons (Regulations on Patient Records, § 8 2019). Information about holding the child still during dental treatment or subjecting the child to other means of restraint can be considered relevant information in dental records for communication between dental health personnel. However, for patients under 16 years of age, parents or caregivers have the legal right to consent on their behalf (Patients and User Rights Act, § 4-4 1999). Thus, the use of restraint can be administered with parental consent and without the child’s assent, meaning that children’s rights (United Nations 1989) are less explicit in law and legal guidelines. Since restraint use is ambiguous in paediatric care, there is a lack of knowledge on how the use of restraint is documented in dental records.

The primary purposes of this study were to examine dental records of Norwegian adolescents’ with and without self-reported history of restraint for information about oral health (DMFT), total scheduled time in the PDS (dental appointments, cancelled and missed appointments), and reluctant behaviour or dental fear and anxiety (DFA). Another purpose was to explore their dental records for information recorded by the dentist concerning the use of restraint.

## Methods

This retrospective study used data from both a cross-sectional study about self-reported history of restraint during dental treatment and the participants written dental records. The data from the cross-sectional study were collected from October to December 2019 and compared with data collected from the dental records from November to December 2020.

The electronic cross-sectional survey was distributed via text message to all 17-year-old adolescents in the PDS in Hordaland, Norway. The PDS in Norway is responsible for individually adapted, free-of-charge follow-up of oral health of children and adolescents aged up to 18 years (Dental Health Service Act, § 1–3 1983). By law, the Norwegian PDS is required to promote the oral health in the population and ensure necessary prevention and treatment (Dental Health Service Act, § 1–2 1983). Most dentists in the Norwegian PDS are non-specialists, and of all dentists, approximately 1% (47) are specialists in paediatric dentistry (Statistics Norway). General dentists and paediatric dentists educated in Norway are not trained in administering restraint. The age group ‘17-year-olds’ were addressed to include people who were still patients in the PDS and could report on their accumulated experiences in the PDS. Although the adolescents were 18-year-olds at the year of the dental record data collection, they were 17-year-olds at the time of the cross-sectional study; therefore, referred to as 17-year-olds. Hordaland County, which includes Bergen, Norway’s second largest city, was in 2019 the third most populated county in Norway (Statistics Norway). The county is mostly rural and sparsely populated outside of the Bergen metropolitan area, which reflects the country. The median household income (711,000 NOK) is quite similar to the median national household income (686,000 NOK) (Statistics Norway). Thus, Hordaland can be regarded representative for epidemiological research in Norway.

### Sample

All 17-year-old participants who participated in the cross-sectional study (*n* = 3305, 52.2%) were invited to participate in this study. Those who provided written informed consent for access to their dental records were eligible for the present study (*n* = 1045).

Based on the cross-sectional data collection, the adolescents were assigned into two groups: one with self-reported history of physical restraints (restraint group) and one group without self-reported history of restraint (non-restraint group). These groups were selected based on the question ‘Have you experienced being held still against your will during dental treatment?’, which was answered by 2560 17-year-olds. All eligible participants in the restraint group were included (*n*_1_ = 26) in this study. The sample size of the self-reported non-restraint group (*n*_2_ = 200, power 0.80 with an effect size of 0.55) was calculated by a statistician. The function ‘random organisation’ in SPSS was used to select 200 participants for the self-reported non-restraint group.

### Data collection and variables

Five elements obtained from the cross-sectional study were used in this study. The first question identified the history of physical restraint (1). The answer *do not know* was counted as *no*. Self-reported history of physical restraint (answer *yes*) was labelled ‘patient-self-reported restraint’. The self-reported age (2) and situation (3) of when physical restraint occurred was measured by ‘Approximately how old were you when/the first time you experienced being physically held still against your will during dental treatment?’, and ‘In what/which situation(s) were you being physically held still during dental treatment?’. Dental fear was assessed using the Children’s Fear Survey Schedule–Dental subscale (CFSS-DS) (4) and a single-item question (5). The self-report version of the CFSS-DS (Cuthbert and Melamed 1982; Gustafsson et al. 2010)) addresses different aspects of dental treatment and is intended to categorise the degree of DFA in children. Each item is scored from 1 (*not afraid at all*) to 5 (*very afraid*), with a total score ranging from 15 to 75. A sum score of > 38 indicates a high DFA (Gustafsson et al. 2010). The single-item question to separate ‘no fear’ from all other levels of dental fear was ‘Are you afraid of dental treatment? (*not at all*, *low degree*, *neither high nor low*, *high degree*, or *very high degree*).

For the 226 participants included in this study, the patients written dental records for the period 2002–2019 were reviewed. All data extractions from the dental records were performed by the first author and a research assistant according to a pre-set protocol. Ten random dental records were double checked to retrieve consistency and no differences were found. Data about oral health and dental treatment, total scheduled time in the PDS, reluctant behaviour and DFA, and recorded use of restraint were collected from the dental records. Table 1 presents an overview of the variables extracted from the cross-sectional study and dental records. The data collected from the dental records had been written by public non-specialist dentists and to a small degree dental hygienists.

**Table 1:** Overview of the variables included in this study

Topic	Variables from the cross-sectional study	Registration (code)
	Patient-self-reported history of physical restraint	No (0), yes (1)
	Age of when restraint had happened (only answered by the yes-responders on the question about physical restraint)	Age 0–17, do not know
	Situational description of the restraint situation (only answered by the yes-responders on the question about physical restraint)	Copied written text
	Patient-self-reported dental fear (CFSS-DS sum score)	≤ 38 (0), > 38 (1)
	Patient-self-reported dental fear (single item)	Not at all/neither high nor low (0), low degree/high degree/very high degree (1)

Topic	Variables from the dental records	Registration (code)
Oral health and treatment	Decayed missing filled teeth (DMFT)	Count (0–28)
	Untreated caries > D_2_	Count
	Dental treatment under conscious sedation	Count
	Dental treatment under general anaesthesia	Count
	Total cancelled/moved appointments (patients’ desire)	Count
Total scheduled time in the PDS	Total missed appointments	Count
	Planned treatment not completed	Count
	Number of therapists (dentists and dental hygienists)	Count
	Habituating the child to dental treatment post-recorded use of restraint	No (0), yes (1), unclear (2)
	Habituating the child to dental treatment	Count
	Reluctant behaviour in child aged 0–5 years	No (0), yes (1), unclear (2)
Reluctant behaviour and/or DFA	Reluctant behaviour during oral examination	No (0), yes (1), unclear (2)
	Reluctant behaviour during dental treatment	No (0), yes (1), unclear (2)
	Description of reluctant behaviour and/or signs of dental fear and anxiety	Copied written text
Restraint	Patient fearful/anxious, written in dental record	No (0), yes (1), unclear (2)
	Restraint registered in dental record	No (0), yes (1)
	Restraint registered in dental record	Count
	Conscious sedation and restraint registered in dental record	No (0), yes (1)

To strengthen validity in the dental record data collection, the words and phrases that could be compatible with reluctant behaviour and/or DFA and the use of restraint were noted and discussed. After assessment in the research group, descriptions of ‘reluctant behaviour and/or DFA’ and ‘restraint’ were operationalised. Restraint was registered when it was explicitly written that the child, for example, had been held still by parents or dental health personnel during dental treatment.

### Statistical analyses

Dental records that missed information from parts of the study period (for instance because of moving to another county or country) were excluded from the analysis. The variables were dichotomized as follows: records with descriptions of the use of restraint were coded 1 and records without restraint descriptions were coded 0. The CFSS-DS was coded 0 for sum scores ≤ 38 and 1 for > 38. The five-point item on DFA was coded 0 for *not at all/neither high nor low* and 1 for *low degree/high degree/very high degree*. Variables coded 2 (unclear) in the data collection were counted as 0 (no) in the analysis.

Statistical analyses were performed using IBM SPSS Statistics for Windows, version 27.0 (IBM, Armonk, NY, USA). Descriptive statistics were conducted using ‘Frequencies’. Mann–Whitney *U* tests were used to compare group differences and Chi-squared tests for independence to indicate variable associations. When the lowest expected frequency in any cell was < 5, the *p* value for Fisher’s exact probability test was reported. The level of statistical significance was set at *p* < 0.05.

### Ethical approval

The study was conducted in accordance with the guidelines of the Helsinki Declaration. The Norwegian Centre for Research Data (#783349/2019) and the County Dental Officer in Hordaland County Municipality (now Vestland) approved this study. Written informed consent was obtained from all individual participants included in the study.

## Results

**Fig. 1:**
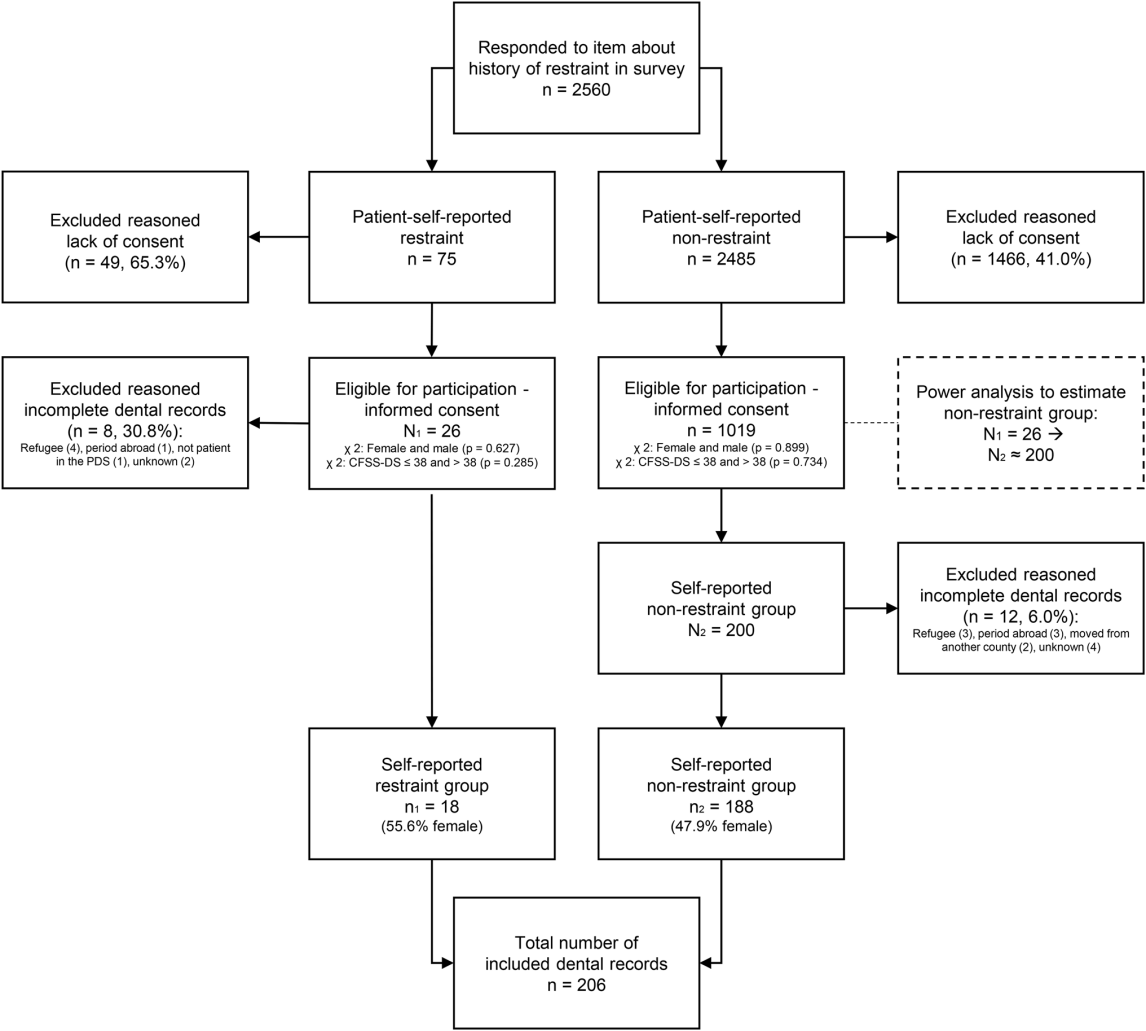
Flow chart of the inclusion and exclusion of participants in the patient-self-reported restraint group (N_1_) and patient-self-reported non-restraint (N_2_) group. All included participants had answered a question concerning history of physical restraint in a cross-sectional survey on restraint in the Public Dental Service (PDS) in Hordaland, Norway (2019) and given informed consent to participate in this study

In total, 69.2% (*n*_1_ = 18) of the self-reported restraint group and 94.0% (*n*_2_ = 188) of the self-reported non-restraint group had complete dental records for the entire period (0–17 years) and were included in the analyses. Figure 1 shows a flow chart of the inclusion and exclusion of participants, including sex distribution in both groups and analysis of participants with and without consent. The self-reported restraint group had more participants with high DFA (22% scored > 38 on the CFSS-DS) than the self-reported non-restraint group (3.7% scored > 38 on the CFSS-DS).

### Oral health and treatment

At the time of data collection, the mean caries experience (DMFT) for the total sample was 3.07. The self-reported restraint group had higher DMFT and more untreated caries (> D_2_) compared with the self-reported non-restraint group (Table 2). Twenty-one (10.2%) patients had experienced treatment(s) with conscious sedation. The distribution of participants was 38.9% (*n* = 7) in the self-reported restraint group and 7.5% (*n* = 14) in the self-reported non-restraint group. There was a significant association between patient-self-reported restraint and history of dental treatment under general anaesthesia (*p* = 0.002).

**Table 2:** Mann–Whitney *U* test results for the differences in the patient-self-reported restraint and non-restraint groups regarding oral health and treatment and total scheduled time in PDS

	Variable collected from dental record	Patient-self-reported restraint group		Patient-self-reported non-restraint group		Statistics (Mann–Whitney *U* Test)
		Median	Mean (SD)	Median	Mean (SD)	
Oral health and treatment	DMFT	4.5	6.94 (6.91)	2.00	2.70 (3.37)	*U* = 2237, *z* = 4.12, *p* < 0.001, *r* = 0.29
	Untreated caries > D_2_	0.00	2.06 (3.65)	0.00	0.16 (0.61)	*U* = 2477, z = 3.31, *p* = 0.001, *r* = 0.23
Total scheduled time in the PDS	Dental appointments (in total)	24.00	26.28 (16.12)	14.00	16.47 (9.01)	*U* = 2284, *z* = 2.46, *p* = 0.014, *r* = 0.17
	Cancelled/moved appointments	5.00	7.00 (7.97)	2.00	2.95 (2.60)	*U* = 2344, *z* = 2.73, *p* = 0.006, *r* = 0.19
	Missed appointments	2.50	3.11 (2.74)	1.00	1.61 (2.24)	*U* = 2387, *z* = 2.99, *p* = 0.003, *r* = 0.21
	Planned treatment not completed	0.5	1.44 (2.12)	0.00	0.13 (0.47)	*U* = 2414.5, *z* = 5.10, *p* < 0.001, *r* = 0.36
	Number of therapists	8.50	11.72 (7.36)	7.00	7.55 (3.93)	*U* = 2287.5, *z* = 2.48, *p* = 0.013, *r* = 0.17
	Habituating the child to dental treatment	0.00	3.50 (6.05)	0.00	0.21 (0.78)	*U* = 2326.5, *z* = 4.41, *p* < 0.001, *r* = 0.31

*SD* standard deviation, *U* Mann–Whitney *U* value, *z* *z* score (standardised test statistics), *p* *p* value, *r* effect size

### Total scheduled time in the PDS

The total scheduled time in the PDS was significantly higher in the self-reported restraint group compared with the self-reported non-restraint group. The self-reported restraint group had more dental appointments, missed appointments, and cancelled appointments. The number of appointments where planned dental treatment was not completed also differed significantly between the two groups. In addition, the total number of therapists involved in the child’s dental healthcare was higher in the self-reported restraint group than in the self-reported non-restraint group. The results are listed in Table 2.

One of the 11 situations of recorded use of restraint was followed up with a new appointment with the intention to habituate the child to the dental situation. Overall, more appointments for habituation to dental treatment were registered in the self-reported restraint group than in the self-reported non-restraint group (Table 2).

### Reluctant behaviour and DFA

There was a significant association between patient-reported dental fear at any level above ‘no fear’ and descriptions of dental fear in the dental records (*p* = 0.007), but there was no association between patient-reported *high* dental fear (CFSS-DS sum score > 38) and records of dental fear (*p* = 0.235).

Reluctant behaviour was registered when the dental record included descriptions such as: refused, protested, unwilling to receive treatment, uncooperative, and reluctant. DFA was registered when the dental record included descriptions such as: anxious, injection/dental fear, dental phobia, terrified, and scared. In the self-reported restraint group, 72.2% had descriptions of reluctant behaviour and 50.0% had DFA descriptions, while in the self-reported non-restraint group, 30.9% had descriptions of reluctant behaviour and 17.2% had DFA descriptions.

There was a significant association between patient-self-reported physical restraint and records of reluctant behaviour in the following situations: children aged 0–5 years (*p* = 0.003), during oral examination at any age (*p* = 0.001), and during dental treatment at any age (*p* = 0.001). For all patients, dentist-recorded reluctant behaviour during examination was significantly associated (*p* < 0.001) with reluctant behaviour during treatment (Table 3).

**Table 3:** Distribution of dental records of reluctant behaviour recorded at oral examination and during dental treatment

	Reluctant behaviour during dental treatment		Total
	Yes	No	
Reluctant behaviour during oral examination			
Yes	24 (53.3%)	21 (46.7%)	45
No	25 (15.5%)	136 (84.5%)	161
Total	49	157	206

### Description of restraint use in dental records

Three of the 206 dental records had descriptions of restraint use. The remaining dental records had no descriptions of restraint. In the self-reported restraint group (*n*_1_ = 18), one patient had dentist-recorded descriptions of restraint. In the self-reported non-restraint group (*n*_2_ = 188), two patients had dentist-recorded descriptions of restraint. Two of the three dental records with restraint descriptions involved conscious sedated. No significant association between patient-self-reported restraint and the dentist-recorded use of restraint was found (*p* = 0.241).

Descriptions of restraint included the following information—Patient 1. Mother holds the patient during oral examination at age 5 years. Father holds the girl during oral examination at age 6 years. This patient reported in the survey that physical restraint occurred when she was 6 years old, where she had a toothache and contacted the dental clinic for help. Patient 2. The patient was held still by guardian to receive rectal Midazolam (conscious sedation) and during dental treatment under conscious sedation at age 4 years. The patient had not reported the restraint experience in the survey and had, therefore, not answered at what age and the situation during which restraint had occurred. Patient 3. Mother holds the patient (conscious sedated) during caries excavation and filling at the age 6 years. The patient had not reported the restraint experience in the survey and had, therefore, not answered at what age and the situation during which restraint had occurred.

## Discussion

The main results of this study were that the adolescents with self-reported history of restraint have poorer oral health, higher total PDS use, and a higher number of descriptions of reluctant behaviour and/or signs of DFA compared with the self-reported non-restraint group. Dental records contained limited information on the use of restraint and did not match the adolescents’ self-reported history of physical restraint.

The intention of this study was to gain knowledge on adolescents’ self-reported experiences and to examine whether variables in their written dental records were different for adolescents with history of restraint during dental treatment compared to adolescents without self-reports of such an experience. The retrospective design prevents from drawing conclusions due to confounders and recall bias. However, results from a prospective cohort study (the Tromsø study) regarding mental health, general health, and well-being indicate that recall is stronger for actual events than for subjective assessments, such as family well-being (Sheikh et al. 2016). In general, the self-reported restraint group had higher DFA compared to the self-reported non-restraint group. Anxious patients may have interest in finding reasons for their anxiety. As such, it is possible that patients with higher DFA scores (CFSS-DS) will ruminate about past experiences during dental treatment, and therefore, report more such experiences than non-anxious peers. It should also be noted, the participants might have, consciously or unconsciously, provided incorrect answers. Still, patients’ own personal experience is valuable information (Beaton et al. 2014).

The results of the present study indicate that the self-reported restraint group had higher DMFT, more untreated caries, more appointments in total, and more missed and cancelled appointments, and more dentists involved compared with the self-reported non-restraint group. These findings are in line with previous studies examining DFA in dental records (Klingberg et al. 1995; Skaret et al. 1999, 2000) and were expected since the self-reported restraint group had a higher percentage with high DFA (> 38 CFSS-DS). Reasons for missed dental appointments might range from forgetfulness to dental phobia. Patients who miss dental appointments should receive customised care (Wang and Aspelund 2009), and as the results of the present study indicate, this should particularly be if they report high DFA and restraint experience. There may be an association between self-reported histories of restraint as a young child and poor oral health at 17 years of age, but this does not mean that the use of restraint was the cause of subsequent poorer oral health and more use of PDS in the future. There are many other variables that could be associated with poor dental health, DFA, and use of dental services, which have complex and multifactorial reasons. For example, dental caries is a multifactorial disease with multiple and complex interactions between environmental, behavioural, and genetic factors. The best predictor of developing caries in the future is the history of past caries experience (Mejàre et al. 2014). Therefore, this study would have been improved if it had included the severity of dental caries in the adolescents when young. Even though no causal conclusions can be made, the treatment and follow-up for the restraint group have been more expensive for the PDS and this should receive attention. The significantly higher number of untreated caries in the self-reported restraint group may indicate that persons with history of restraint also face challenges in receiving dental care. When restraint is used, psychosocial challenges in the dental situation should be addressed during follow-up appointments to help the patient overcome possible negative feelings.

Most descriptions of reluctant or fearful patients were found in the dental records of the self-reported restraint group. This supports the results of a cross-sectional study that showed that patients with self-reported history of restraint have significantly higher dental fear compared with those who had no such experience (Aarvik et al. 2022). Further, Sturmey reported that fearful patients have a higher risk of being restrained (Sturmey 2015). Many patients were described in their dental records as uncooperative, reluctant, or unwilling to receive treatment. These descriptions mirror dental behaviour management problem(s) (BMP) in young patients (Klingberg and Broberg 2007). Klingberg and Broberg defined BMP as ‘a collective term for *uncooperative* and disruptive behaviours, which result in delay of treatment or render treatment impossible, regardless of the type of behaviour or its underlying mechanism(s)’ (Klingberg and Broberg 2007). This present study indicates that the self-reported restraint group are described as more reluctant and/or fearful.

Although we could not determine whether the patients were fearful or had BMP even before the restraint situation, negative experiences are a well-known aetiological cause for the development of DFA (Klingberg and Broberg 2007; Klingberg 2008; Locker et al. 2001; Milsom et al. 2003; Ost and Hugdahl 1985; Seligman et al. 2017; Ten Berge et al. 2002; Åstrøm et al. 2021), and there is reason to hypothesise that experiencing restraint during dental treatment is a negative experience which can influence DFA. Painful dental treatment is one of the most frequently mentioned causes of DFA and BMP, especially in combination with a feeling of lack of control (Seligman et al. 2017). The dental records of restraint had no information about painful treatment or inabilities to achieve profound analgesia, but this does not mean that it was not present. In this study, the only oral pathology measured was caries. Conditions such as Molar Incisor Hypomineralisation with problems concerning pain/sensitivity could also be one of the possible factors associated with the development of DFA (Jälevik et al. 2021). Further, medical and psychological conditions such as autism, general fear, and child temperament have been reported to be associated with occurrence of DFA and BMP (Blomqvist et al. 2014; Klingberg 2008; Seligman et al. 2017). These conditions can be anticipated to influence such a child’s emotional response to restraint.

In the analysis of participants who provided consent and those who did not, we found no associations for sex and high DFA (> 38 CFSS-DS) (Fig. 1). The reasons for why the noticeably lower percentage of the self-reported restraint group consented to participate in the present study are unknown. If a child has had negative feelings and received verbal appraisals from their dentist for reluctant or uncooperative behaviour, feelings of shame may be prominent (Nathanson 1994). Other potential reasons not to consent can include no interest in the topic, scepticism, or unwillingness to give identifiable information. The self-reported restraint group also had the highest percentage share of adolescents with incomplete dental records (excluded from analyses), and the majority of incomplete records belonged to refugees or persons who had lived abroad. This might imply that some of the self-reported restraint situations during dental treatment have occurred outside the Norwegian PDS. Rønneberg et al. discussed how dentists’ educational backgrounds might influence the prevalence and acceptance of the use of restraint (Rønneberg et al. 2017). Thus, this finding might indicate that dentists should be especially aware of patients with unknown dental history regarding behavioural objectives.

The identified discrepancy between patient-self-reports and dental records can be problematic because being subjected to restraint can cause psychological, social, and developmental burdens for a child (Amos 2004; Diseth 2006; Sturmey 2015). Sparsely written dental records regarding behavioural objectives may be the reason for this discrepancy. A one-sided focus on oral diagnosis and operative treatment in the dental records may not benefit the child and may not be in accordance with the legal regulation of medical records: health personnel are required to record sufficient information to treat the patient (Health Personnel Act, § 40 1999). Given that the parent or caregiver consents to the treatment in which a child can experience restraint, then legally, the practise is, by Norwegian law, not considered as formal restraint (Patients and User Rights Act, § 4-4 1999). Hence, it can be considered unnecessary to document restraint in the dental record, which may explain several discrepancies in documentation. The difference between patient-self-reports and the dentist's written reports of the treatment might be explained by DFA, since patients with DFA might be better aware of restraint, while a non-DFA patient would rather perceive restraint as support or guidance. In general, notes from dental records seldom give a complete picture of the treatment situation (Klingberg et al. 1995). Since both public and paediatric dentists relate to the practice of restraint with feelings of negativity and professional failure (Aarvik et al. 2021; Marty et al. 2020), dentists may simply fail to document the use of restraint despite knowing that they have used restraint. Without well-defined guidelines on the use of restraint, a dentist must individually assess whether restraint is the method of choice (Aarvik et al. 2021; Marty et al. 2020). On the 31st of March 2022, new national guidelines for dentists treating children and adolescents in Norway were published and the use of restraint was included (Norwegian Directorate of Health 2022). Dentists are recommended to only use restraint as a last resort method after a thorough assessment, consulting a paediatric dentist if necessary. The use of restraint shall be documented in the patients’ dental record (including justification, procedure, and cooperation with the child/parents) and have a follow-up with the child within a week to ensure that the child receives good follow-up in the future (Norwegian Directorate of Health 2022). In this study, the fact that the self-reported restraint group had significantly more therapists than the self-reported non-restraint group underscores the importance of comprehensive recording so that dentists involved in the patient’s dental care are informed and can customise the care provided.

Most recorded descriptions of restraint were related to treatments where the young patients were conscious sedated. Similarly, a qualitative study of Norwegian non-specialist dentists indicated that the use of restraint often is legitimised when applied in combination with conscious sedation (Aarvik et al. 2021). In 2017, Rønneberg et al. reported that 12% of dentists in the Norwegian PDS used restraint to administer acute dental treatment to young children (Rønneberg et al. 2017). Furthermore, 50% would give a new appointment with conscious sedation. The study did not mention if the sedated treatment could include restraint. How restraint occurs during dental treatments in combination with conscious sedation in the Norwegian PDS should be explored in prospective studies.

### Limitations

The results of retrospective designs must be interpreted with caution. The small sample size with the possibility of selection bias is a weakness of this study. Of the 17-year-olds in the target population, 52.2% participated in the cross-sectional study, and of those, only 31.6% (1045/3305) gave informed consent for participation in this present study. Further, several dental records were excluded from the analyses due to incomplete dental records. This limitation must be considered when the results are interpreted. However, the mean DMFT score in this study (3.07) is similar to the mean DMFT scores for 18-year-olds in Vestland county municipality (former Hordaland) and Norway in general (3.00) (Statistics Norway) which supports the representativeness of the current sample. The small sample in the self-reported restraint group made it necessary to include a higher number of participants in the self-reported non-restraint group. Therefore, subgroup analysis of for example sex differences was not possible.

The study gives no information about the patients’ oral health and self-assessed DFA at the time before their self-reported history of restraint during dental treatment and cannot conclude on causal relationships. Including the parents’ DFA would be valuable since DFA may be learned by modelling, listening to others, or be a result of heredity and personality traits (Beaton et al. 2014). In addition, the parent’s evaluation of their child’s experiences in the PDS and considerations on the aetiology of the child’s DFA would be valuable. Another limitation is that the patients’ somatic and psychological health was not assessed. Nevertheless, this study is the first to examine patient-self-reported history of restraint compared with dentist-recorded restraint and provides new knowledge in the field of paediatric dentistry.

## Conclusion

Considering the limitations of the present study, it has been shown that the adolescents with self-reported history of restraint had higher DMFT, higher scheduled time attending the PDS and had more descriptions of reluctant behaviour and/or signs of DFA compared with the self-reported non-restraint group. The dental records written by non-specialist dentists had sparsely written descriptions regarding restraint, and the comparisons showed that patient-self-reported restraint was not consistent with dentist-recorded restraint. Dentists should strive to, in addition to the administered dental treatment, address behavioural objectives in the dental records. Due to the small numbers included in the study, conclusions cannot be drawn, and negative consequences of restraint should be addressed in future prospective studies.

## Data Availability

The data that support the findings of this study are available from the corresponding author upon reasonable request.
